# Engineering of CHO Cells for the Production of Recombinant Glycoprotein Vaccines with Xylosylated *N*-glycans

**DOI:** 10.3390/bioengineering4020038

**Published:** 2017-04-28

**Authors:** Grit Sandig, Hans Henning von Horsten, Lars Radke, Véronique Blanchard, Marcus Frohme, Christoph Giese, Volker Sandig, Stephan Hinderlich

**Affiliations:** 1 Laboratory of Biochemistry, Department of Life Sciences and Technology, Beuth University of Applied Sciences Berlin, Seestrasse 64, 13347 Berlin, Germany; Grit.Sandig@htw-berlin.de; 2 Department of Life Science Engineering, HTW Berlin University of Applied Sciences, Wilhelminenhofstraße 75a, 12459 Berlin, Germany; HansHenning.vonHorsten@htw-berlin.de; 3 Molecular Biotechnology and Functional Genomics, Technical University of Applied Sciences Wildau, Hochschulring 1, 15745 Wildau, Germany; lradke@th-wildau.de (L.R.); mfrohme@th-wildau.de (M.F.); 4 Institute of Laboratory Medicine, Clinical Chemistry and Pathobiochemistry, Charité Medical University Berlin, Augustenburger Platz 1, 13353 Berlin, Germany; veronique.blanchard@charite.de; 5 ProBioGen AG, Goethestrasse 54, 13086 Berlin, Germany; christoph.giese@probiogen.de (C.G.); volker.sandig@probiogen.de (V.S.)

**Keywords:** CHO, glycoengineering, respiratory syncytial virus, vaccine, xylose

## Abstract

Xylose is a general component of *O*-glycans in mammals. Core-xylosylation of *N*-glycans is only found in plants and helminth. Consequently, xylosylated *N*-glycans cause immunological response in humans. We have used the F-protein of the human respiratory syncytial virus (RSV), one of the main causes of respiratory tract infection in infants and elderly, as a model protein for vaccination. The RSV-F protein was expressed in CHO-DG44 cells, which were further modified by co-expression of β1,2-xylosyltransferase from *Nicotiana tabacum*. Xylosylation of RSV-F *N*-glycans was shown by monosaccharide analysis and MALDI-TOF mass spectrometry. In immunogenic studies with a human artificial lymph node model, the engineered RSV-F protein revealed improved vaccination efficacy.

## 1. Introduction

Glycosylation is one of the most important post-translational modifications. Proteins can be decorated with glycans, which are linked either with asparagine residues via an *N*-glycosidic linkage (*N*-glycans) or with serine or threonine residues via an *O*-glycosidic linkage (*O*-glycans) [1]. The majority of *N*- and *O*-glycans consists of six different monosaccharides, namely mannose, galactose, *N*-acetylglucosamine (GlcNAc), *N*-acetylgalactosamine (GalNAc), fucose and sialic acids. Xylose is a rare monosaccharide component of *O*-glycans [2], and it is part of the linker between glycosaminoglycans and proteins [3]. Furthermore, pentose, together with glucuronic acid, is one of the two monosaccharide components of a heteropolymer linked to α-dystroglycan in muscle cells [4], where it mediates its interaction with the extracellular matrix component laminin. In *N*-glycans xylose is only found in plants and helminth [5,6]. There it is usually β1,2-linked to the central mannose residue of the *N*-glycan core.

For mammals, xylosylated *N*-glycans are alien structures. Parenteral introduction of glycoproteins carrying this kind of *N*-glycans into mammals therefore leads to strong immune reactions [5,7]. For the production of recombinant therapeutic glycoproteins in plant cells immunogenicity of the *N*-glycans is therefore an issue [7]. To date, plant cell lines used for recombinant glycoprotein productions are engineered by knock-out of the respective β1,2-xylosyltransferase [8,9]. However, the metabolic pathway for the synthesis of xylose and its activated nucleotide sugar UDP-xylose, respectively, is present also in mammalian cells [10]. UDP-xylose is synthesized from UDP-glucose in two steps; first by oxidation of the C-6 atom, resulting in UDP-glucuronic acid. The same C-6 atom is then decarboxylated, and the pentose xylose is generated, which is bound to UDP in the furanose form. UDP-xylose is then transported to the Golgi apparatus, where it serves as substrate for xylosyltransferases. 

Altered glycans may increase or modulate the immunogenicity with potential benefits for recombinant glycoprotein vaccines [11,12]. Introduction of core β1,2-xylosylation is one promising approach [13]. In this study we have used a protein of the human respiratory syncytial virus (RSV) as a model vaccine. RSV is the main cause of lower respiratory tract infections in infants and the elderly, and also affects high-risk adults [14,15]. Although several strategies, including use of vaccines and therapeutic antibodies, have been tested for treatment of RSV infections in the last few decades, no highly efficient prevention therapy is presently available [16]. RSV vaccines based on recombinant proteins could benefit from the two major surface proteins RSV-F (fusion protein) and RSV-G (glycoprotein). In this study we used a truncated form of RSV-F, which only contains the extracellular part. RSV-F has five *N*-glycosylation sites, whereby two of them are released together with a 27 amino acid peptide by intracellular cleavage by the Golgi protease furin [17]. Mature RSV-F therefore consists of two disulfide-bridged polypeptide chains with *N*-glycans at the positions Asn-23, Asn-66 and Asn-497 [17,18,19]. The recombinant RSV-F protein was produced in glycoengineered CHO DG44 cells, co-expressing the β1,2-xylosyltransferase (XylT) from *Nicotiana tabacum*. We were able to show that RSV-F from glycoengineered cells contains xylosylated *N*-glycans, and that this recombinant vaccine, compared to RSV-F with non-xylosylated *N*-glycans, displays stronger immunogenicity in a human artificial lymph-node (HuALN) model. 

## 2. Materials and Methods

### 2.1. Materials

CDC4 medium with 4.5 g/L glucose was obtained from ProBioGen AG, Berlin, Germany, Dulbecco’s phosphate buffered saline (DPBS) from PAN-Biotech GmbH (Aidenbach, Germany), adenovirus expression medium (AEM) from Life Technologies GmbH (Darmstadt, Germany), fetal calf serum (FCS) superior from Merck Millipore (Darmstadt, Germany). Unless otherwise stated, all chemicals were purchased from Carl Roth GmbH + Co. KG (Karlsruhe, Germany) or Sigma-Aldrich GmbH (Taufkirchen, Germany).

### 2.2. Cell Culture

Standard cultivation of all cells was performed in CDC4 medium supplemented with 6 mM l-glutamine, 50 ng/mL IGF and 100 µg/mL penicillin/streptomycin (100 U/mL) within a 8% CO_2_ atmosphere at 36.5 °C. Stably RSV-F-expressing CHO-DG44 cells and additional XylT expressing CHO-DG44 cells were cultivated under serum-free conditions in CDC4 medium supplemented with 6 mM l-glutamine, 1% penicillin/streptomycin (100 U/mL, 100 µg/mL) and insulin-like growth factor (50 ng/mL).

### 2.3. Construction of Expression Vectors

The RSV-F cDNA was constructed corresponding to Ternette et al. [20], with the exception that the signal sequence was replaced by the mellitin signal sequence (according to Acc. No. P01501; MKFLVNVALVFMVVYISYIY). It is followed by the ectodomain of the RSV-F protein (amino acids 26 to 530; according to Acc. No. EF566942), a Factor Xa cleavage site (IEGR), and a GSGS linker fused to a 6xHis-tag (HHHHHH). The gene was codon optimized for *Cricetulus griseus* and synthesized with flanking EcoRI and BamHI restriction by Gene Art (Regensburg, Germany). The gene was cloned into the EcoRV restriction site of the vector pBGGPEX1 (ProBioGen AG, Berlin, Germany) by EcoRI/BamHI digestion, and followed by DNA polymerase Klenow (Roche, Mannheim, Germany) treatment resulting in the pBGGPEX1-RSV-F vector.

The gene of *Nicotiana tabacum* XylT (Acc. No. AJ627182) was synthetized by Gene Art (Regensburg, Germany) with a codon-optimized sequence for *Cricetulus griseus*, flanked by BglII/EcoRI restriction sites. The gene was cloned into the expression vector EF2flag neo (ProBioGen) using the BglII/EcoRI restriction sites resulting in the vector EF2flag XylT. Plasmids were prepared by the QIAprep^®^ Spin Miniprep kit (Qiagen, Hilden, Germany) and EndoFree^®^ Plasmid Maxi kit (Qiagen).

### 2.4. Transfection of CHO Cells

The CHO-DG44 cells were transfected with pBGGPEX1-RSV-F vector by electroporation using the Neon^®^ Transfection system (Thermo Fisher, Schwerte, Germany). Selection of transfected has been carried out for 17 days in serum-free C8862 medium supplemented with puromycin and methotrextate (MTX). The resulting CHO RSV-F cells were additionally transfected with the EF2flag XylT vector by lipofection using the Freestyle Max Reagent (Thermo Fisher) in Optipro medium (Thermo Fisher). CHO-F-XylT clones were selected with Neomycin (G418). The expression of the RSV-F protein has been verified by western blot analysis using a Penta-His HRP antibody (Qiagen, 1:2000). The expression of the XylT was detected on the transcription level by RT-PCR with specific primers (forward 5′-GAGAACCACCACGACAAC-3′, reverse 5′-CTGTTCCTCGTTGGACAG-3′). The resulting PCR product of 1077 bp was visualized by agarose gel electrophoresis.

### 2.5. Protein Production

Serum free fed-batch culture of transfected CHO cells producing modified RSV-F protein was carried out in 50 mL bioreactor tubes containing CD-C4 growth medium supplemented with l-glutamine. Bioreactor tubes were inoculated at a starting cell density of 4 × 10^5^ cells/mL and incubated at 146 rpm, 37 °C, 8% CO_2_. Performance of the fed-batch cultures was monitored for 14 days and supernatant containing the RSV-F protein was harvested after declining of cell viability down to 75%. The supernatant was analyzed for RSV-F production by western blotting. 

### 2.6. Purification of Proteins

RSV-F protein purification was performed by a one-step purification using a 5 mL Ni-NTA cartridge (Machery & Nagel, Düren, Germany). 250 mL of the sterile filtered supernatant from batch production was twice concentrated using an Amicon Filter device (300 mL) and diluted in binding/washing buffer (20 mM sodium phosphate, 0.5 M NaCl, 20 mM imidazole, pH 7). After equilibration of the column with 5 volumes binding buffer the sample was loaded and the column washed with 10 volumes of binding/washing buffer. Protein was eluted in one step by 5 mL elution buffer (20 mM sodium phosphate, 0.5 M NaCl, 500 mM imidazole, pH 7), dialysed against PBS, sterile filtered and stored at 4 °C. Protein concentration was quantified by BCA protein assay for total quantification and RSV-F proteins were analyzed by western blotting.

### 2.7. High-pH Anion-Exchange Chromatography with Pulsed Amperometric Detection (HPAEC-PAD) Monosaccharide Analysis

20 µg of purified proteins was hydrolyzed in 2 M trifluoroacetic acid (TFA) for 4 h at 100 °C. A blank sample was used as negative control. As internal standards 200 pmol 2-deoxy-D-ribose (DR), d-fructose (Fruc) and d-melibiose (Mel) (each from Sigma-Aldrich, Taufkirchen, Germany) were used. As external standards a set of monosaccharides, including 100 pmol L-fucose (Fuc), d-arabinose (Ara), d-galactosamine (GalN), d-galactose (Gal), d-glucosamine (GlcN), d-glucose (Glc), d-Xylose (Xyl) and d-Mannose (Man), was used and run prior to the protein samples. HPAEC-PAD was performed on an ICS-3000 Ion Chromatography System (Thermo Fisher) using a Dionex CarboPac^®^ PA200 column. Monosaccharides were separated by isocratic 2.25 mM NaOH elution while post-column addition of 200 mM NaOH provided the conditions for pulsed amperometric detection. 

### 2.8. Release and Separation of N-Linked Glycans

Tryptic digestion was performed twice (for 4 h at 37 °C and overnight, respectively) using 2.5 µg trypsin (Sigma-Aldrich) per 30 µg of glycoprotein. After trypsin inactivation, samples were treated with 0.5 U of *N*-glycosidase F from Flavobacterium meningospeticum (Roche Diagnostics, Mannheim, Germany) and incubated at 37 °C. After 4 h additional enzyme was added, the sample was incubated overnight followed by inactivation (5 min, 95 °C). *N*-Glycans were separated from the peptide fraction using reversed-phase C18 cartridges and a subsequent desalting step on graphitized cartridges (Grace Davison Discovery Sciences, Worms, Germany) as described before [21]. Purified *N*-glycans were lyophilized and stored at −20 °C.

### 2.9. Permethylation and Matrix-Assisted Laser Desorption/Ionization Time-of-Flight (MALDI-TOF) Mass Spectrometry

Prior to mass spectrometric (MS) analysis, *N*-glycan samples were permethylated in order to stabilize sialic acids and to improve the efficiency of positive ion formation [22]. The derivatization procedure followed standard protocols of the solid sodium hydroxide technique [22,23] with minor modifications. Incubations were carried out under continuous shaking at room temperature. The iodomethane reaction was stopped by the addition of chloroform and subsequent washing steps with water until achieving a neutral pH of the aqueous phase. 

For MALDI-TOF-MS analysis dried permethylated *N*-glycans were dissolved in 75% (v/v) acetonitrile in water and mixed with super-dihydroxybenzoic acid (sDHB) matrix (Sigma-Aldrich). Recording of mass spectra on an Ultraflex III MALDI-TOF/TOF spectrometer (Bruker Daltonik, Bremen, Germany) and subsequent data processing was realized as reported previously [24]. Structures were assigned to related peaks according to the Glycoworkbench 2.0 database, or constructed by the same software if not available. Fragmentation analysis was performed by the integrated Lift method of the mass spectrometer. Fragment sizes were compared to the theoretically determined size of the glycans using Glycoworkbench 2.0. Schematic representation of glycan structures are according to the symbol nomenclature of the Consortium for Functional Glycomics [25]: green circle, mannose; yellow circle, galactose; blue square, GlcNAc; yellow square, GalNAc; red triangle, fucose; purple diamond, *N*-acetylneuraminic acid; star, xylose.

### 2.10. Cytokine and Gene Expression Analysis

Stimulation experiments were performed in a HuALN (ProBioBen AG; [26,27]) In brief, RSV-F and RSV-F Xyl+ were cultivated in the presence of CD14(-) cells and mature dendritic cells prior to addition of PBMC and matrix. HuALN reactors were run for 28 days and re-stimulation took place at days 7, 14 and 21. Cell culture supernatants were taken daily and analyzed with a bead-based multiplexed immune assay (Luminex^®^ technology, Thermo Fisher). A custom Bio-Plex^®^ Express Aassay (Bio-Rad, München, Germany) was used to quantify the six analytes IL-2, IL-4, IL-10, GM-CSF, IFN-γ and TNF-α. The assay was performed in duplicates as described by the manufacturer, but conducted automated on a pipetting robot (Freedom Evo 200; Tecan, Crailsheim, Germany). Quantification of samples based on a logistic 5-point regression method using standard curves with 8 point 4-fold dilution series with analyte-specific concentration ranges. Error limits of data more than twice as much as the detection limit were below 20%.

To investigate the first response of the immunologic reaction comprehensively, gene expression was analyzed with a Human Immunology v2 nCounter^®^ Gene Expression assay (NanoString^®^ Technologies, Seattle, WA, USA) covering 579 immunology related genes. Therefore, PBMC of healthy donors were stimulated for 48 h with antigen-specific mDC and the according proteins, or were left untreated (negative control). Total RNAs of cells were extracted with a High Pure RNA Isolation Kit (Roche Diagnostics). Concentration and purity of total RNAs were controlled with a Nanodrop 1000 (Thermo Fisher) using the spectral absorption quotients 260/230 and 260/280. RNA Quality Numbers (RQN) were determined on a Fragment Analyzer^™^ (Advanced Analytical Technologies, Heidelberg, Germany) with a DNF-472 High Sensitivity RNA Analysis Kit, using extended runtimes of 60 min per sample to be able to detect any remaining genomic DNA contaminations. The nCounter technology [28] is a multiplexed method that quantifies mRNA on single molecule level by using fluorescent barcoding probes and is described in detail in [29]. NanoString experiments were performed according to the manufacturer protocol. Due to slight fragmentation of RNA increased amounts of input material (150 ng) were used in the experiment. RNAs were quantified with Qubit^®^ RNA HS AssayKit (Thermo Fisher), immediately before the nCounter experiment was conducted. The nSolver^™^ Analysis Software 3.0 (NanoString^®^) was used to perform data handling, including automated background subtraction, spike-in-control normalization and reference gene normalization. Furthermore, datasets from duplicates were grouped and fold change estimates were calculated by building ratios with the unstimulated negative control. Since analysis could be performed in duplicates only, somewhat higher p-values were accepted to keep data sets for broad immunologic parts descriptive.

## 3. Results and Discussion

### 3.1. Co-Expression of RSV-F and XylT in CHO DG44 Cells

**Figure 1:**
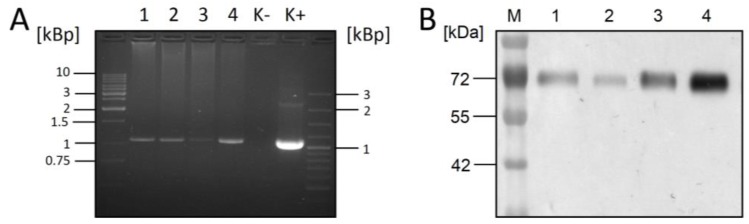
Detection of XylT mRNA and RSV-F-protein in four different CHO-XylT clones. (**A**) XylT mRNA was detected by RT-PCR. Clones 1–4 were compared to non-transfected CHO cells as negative control (K−), and the E2Fflag/XylT vector as RT-PCR template as positive control (K+); (**B**) Maintenance of RSV-F expression in clones 1–4 was verified by western blotting using anti-His antibody.

For expression of the soluble version of RSV-F, the synthetic gene consisting of the extracellular domain of RSV-F equipped with the mellitin signal peptide and C-terminally linked with a factor X cleavage site followed by a hexa-His tag was cloned into the pBGGPEX1 vector and transfected into CHO DG44 cells. The melitin signal peptide is commonly used in insect cells but also functions well in mammalian cells (ProBioGen AG, unpublised). Transfectants were selected with puromycin and MTX and stable clones were generated. SDS-PAGE analysis of cell supernatant displayed a 72 kDa band, indicating successful production of the full-length RSV-F protein. Next, a codon-optimized cDNA of XylT was generated as an artificial gene and cloned into the E2Fflag vector. CHO DG44/RSV-F cells were transfected, selected with Neomycin (G418) and stable cell clones were isolated. Four of these clones were analyzed by RT-PCR for XylT mRNA, and by Western blot for RSV-F (Figure 1). All four clones revealed expression of XylT and maintained RSV-F protein expression, indicating successful co-expression of both proteins. RSV-F protein derived from these clones was labeled “RSV-F Xyl+”.

### 3.2. Monosaccharide Analysis

**Figure 2:**
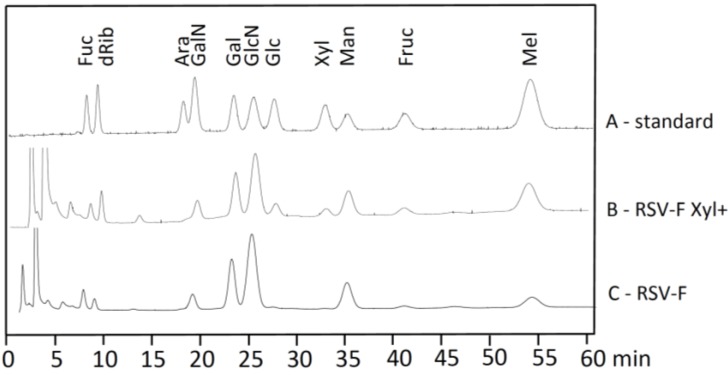
Monosaccharide composition of RSV-F glycans analyzed by HPAEC-PAD. Peaks were compared to standard monosaccharides (A). Xylose could only be found in RSV-F Xyl+ (B) and not in RSV-F (C).

RSV-F and RSV-F Xyl+ were purified from cell culture supernatants by Ni-NTA affinity chromatography; 50 mL of supernatant resulted in about 11 mg of protein with a purity higher than 98%. 25 to 50 µg of protein was subjected to monosaccharide analysis by HPAEC-PAD. Samples were treated with strong acid hydrolysis, leading to the cleavage of acetyl groups from amino sugars, and used for chromatographic separation of monosaccharides in the presence of standards (Figure 2). Chromatograms for RSV-F showed the typical monosaccharide composition of *N*-glycosylated proteins. Monosaccharides from RSV-F Xyl+ displayed a comparable composition, but an additional peak at about 34 min, which could clearly be assigned to xylose when comparing to standards. The peak at about 19 min in RSV-F and RSV-F Xyl+ samples might indicate the presence of GalN, in other words GalNAc before hydrolysis, which is a typical monosaccharide of O-glycosylation. Gruber and Levine [17] previously speculated about the presence of O-glycans in RSV proteins in 1985. However, there is no experimental proof for O-glycosylation of RSV-F to date. The peak for Glc, which is larger in the RSV-F Xyl+ sample compared to the RSV-F sample, does not really indicate O-glcosylation, suggesting more of a typical contamination in this kind of analysis. Finally, O-glycans contain only small amounts of xylose, which could not explain the amount of this monosaccharide found in RSV-F Xyl+ samples. 

### 3.3. Analysis of N-glycans of RSV-F Proteins

**Figure 3:**
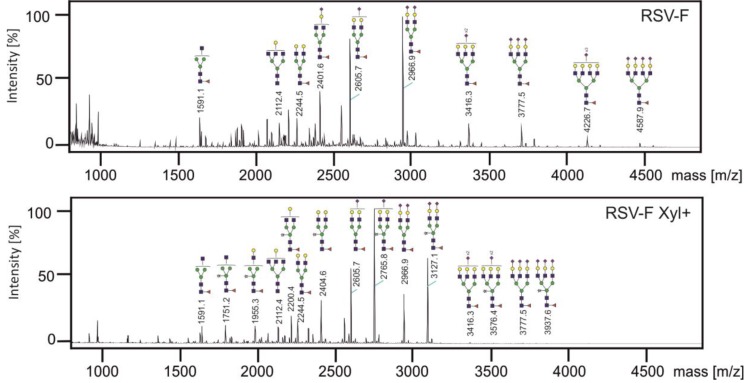
Mass spectrometric analysis of RSV-F *N*-glycans. *N*-glycans were prepared from RSV-F (upper panel) and RSV-F Xyl+ (lower panel), permethylated and analyzed by MALDI-TOF-MS. Structures were assigned to related peaks; xylosylated structures, which are not found in the database, have been constructed using Glycoworkbench 2.0.

The *N*-glycans of both proteins were released by PNGase F, permethylated to allow detection of sialic acid, and analyzed by MALDI-TOF mass spectrometry (Figure 3). For RSV-F five biantennary, three triantennary and two tetraantennary complex *N*-glycans could be found. Most of the *N*-glycans are core-fucosylated and highly sialylated. This *N*-glycan pattern is typical for recombinant glycoproteins from CHO cells [30]. For RSV-F Xyl+ seven *N*-glycans of RSV-F could regained. Furthermore, eight new *N*-glycans could be identified, which carried an additional xylose residue. These data show that the xylose, which was found by monosaccharide analysis, is integrated into *N*-glycans by the action of heterologously expressed XylT. Quantification of *N*-glycans revealed that the biantennary xylosylated *N*-glycans are predominant compared to their non-xylosylated counterpart. On the other hand, reduction or disappearance of tri- and tetraantennary *N*-glycans could be observed. This might be due to strong activity of XylT in the medial Golgi, where branching of *N*-glycans is determined, and the respective GlcNAc transferases IV and V for the formation of the third and fourth antenna [31] have reduced activity by competing with XylT. 

**Figure 4:**
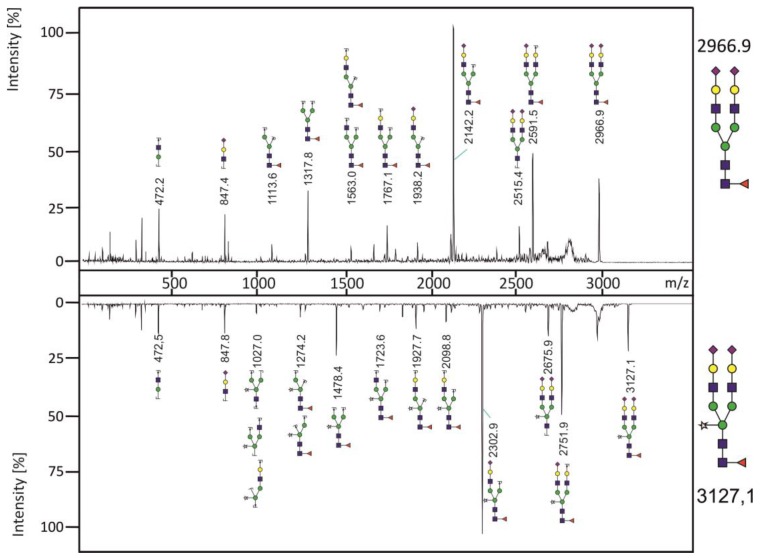
Fragmentation analysis of permethylated *N*-glycans. Fragments were obtained from the peaks m/z 2966.9 (RSV-F) and m/z 3127.1 (RSV-F Xyl+). Note the diagnostic mass differences of 160.1 Da for xylose.

MALDI-TOF analyses (Figure 3) suggest the incorporation of xylose in the *N*-glycans isolated from RSV-F Xyl+. We therefore performed fragmentation analysis to confirm and localize the presence of xylose by comparing the peak m/z 3127.1 with its non-xylosylated counterpart m/z 2966.9 (Figure 4). The spectrum of the latter shows characteristic fragment ions of a biantennary complex *N*-glycan. The fragmentation pattern of the xylosylated *N*-glycan m/z 3127.1 reveals an additional mass of 160 Da of all fragments containing the core mannoses, correlating to the mass of an additional xylose moiety. These data indicate that xylose is bound to the central core mannose, and most likely in a β1,2-linkage, as it is catalyzed by *Nicotiana tabacum* XylT in its original target organism. Similar fragment patterns were obtained for peaks m/z 2765.8 vs. non-xylosylated m/z 2605.7 and peaks m/z 2404.6 vs. non-xylosylated m/z 2244.5 (data not shown). Taken together, we have shown that heterologous expression of *Nicotiana tabacum* XylT results in formation of xylosylated complex *N*-glycans in CHO cells. We have detected also sialylated and triantennary *N*-glycans which does not occur in plants [6], with a core xylose. The action of XylT in CHO cells is therefore independent from the *N*-glycan structure. 

### 3.4. Stimulation of Immune Cells with RSV-F Proteins

**Figure 5:**
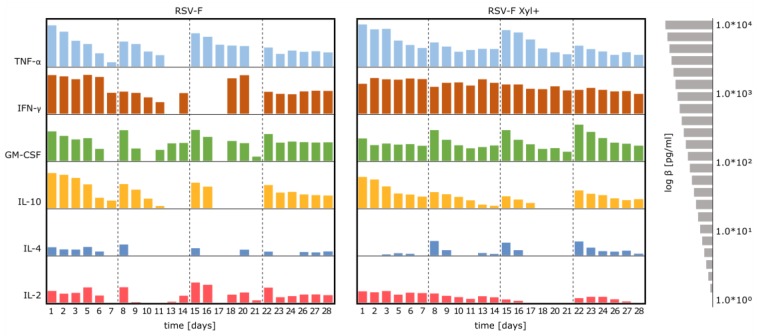
Cytokine secretion pattern in cell culture supernatants of long-term stimulation experiments. PBMCs were stimulated with RSV-F Xyl+ (right) and wildtype RSV-F protein (left) and the according antigen-specific mDC. Concentrations are log10 transformed. (Re-)stimulation took place at day 0, 7, 14 and 21 (dotted line). Supernatants were collected on a daily basis (exceptions at day 4, 12 and 19).

In order to investigate potential immune activation of RSV-F proteins, HuALN reactors were run in the presence of RSV-F and RSV-F Xyl+. Cytokine concentrations were detected in the collected cell culture supernatants with Bio-Plex Express Kits using an automated liquid handling robot (Figure 5). Cytokine expression of the immune cells clearly responded to the time-points of (re-)stimulation in both experiments. In general, cytokine secretion is stronger or more accentuated by cells stimulated with RSV-F Xyl+. IFN-γ levels are substantially high, and twice as high after restimulation. TNF-α concentration peaks after restimulation before returning to a basic level, leading to an overall strong pro-inflammatory response. This is in concordance with the lower level of anti-inflammatory IL-10 (603, 27, 11 and 38 pg/mL, respectively, at days after (re-)stimulation), compared to RSV-F (1342, 140, 184 and 114 pg/mL, respectively). Taken together, these data demonstrate a strong activation of Th1 cells. Besides the inhibiting effect of IL-10 on Th1 cytokines it enhances B-cell survival, proliferation and antibody production. In connection with IL-4, which is more strongly provoked by RSV-F Xyl+ (concentrations are 2.0-fold, 3.1-fold and 8.1-fold higher at days of restimulation), there is evidence for a potent Th2 and humoral response as well. IL-2 levels induced by stimulation with xylosylated RSV-F are continuously present in the first two weeks. However, IL-2 levels are relatively low for both vaccines, compared to studies with other stimulants [32]. Finally, GM-CSF peaks are higher (e.g., 6.9 fold and 9.0 fold higher at day 22 and 23) in the days after restimulation for cells treated with RSV-F Xyl+, indicating an enhancing effect on the cellular response and adaptive immunity, since monocytes can mature into dendritic cells.

In addition to the long-term cultivation in the HuALN reactors, stimulation experiments were conducted to investigate the immunologic first response in high detail on genetic level. PBMCs were stimulated with the vaccine candidates and the respective mDC for 48 h in duplicates. mRNAs were extracted from harvested cells. Concentrations between 16.8 and 42.2 ng/µL, and sufficient purity were obtained, with exception of one sample. Slightly increased concentrations of input material were therefore used in nCounter experiments. The automatic quality control within the nSolver Software was passed by all samples. Due to their high coefficient of variation (>75%) within the analyzed samples GAPDH, OAZ1 and TUBB were excluded from the panel of used reference genes.

**Figure 6:**
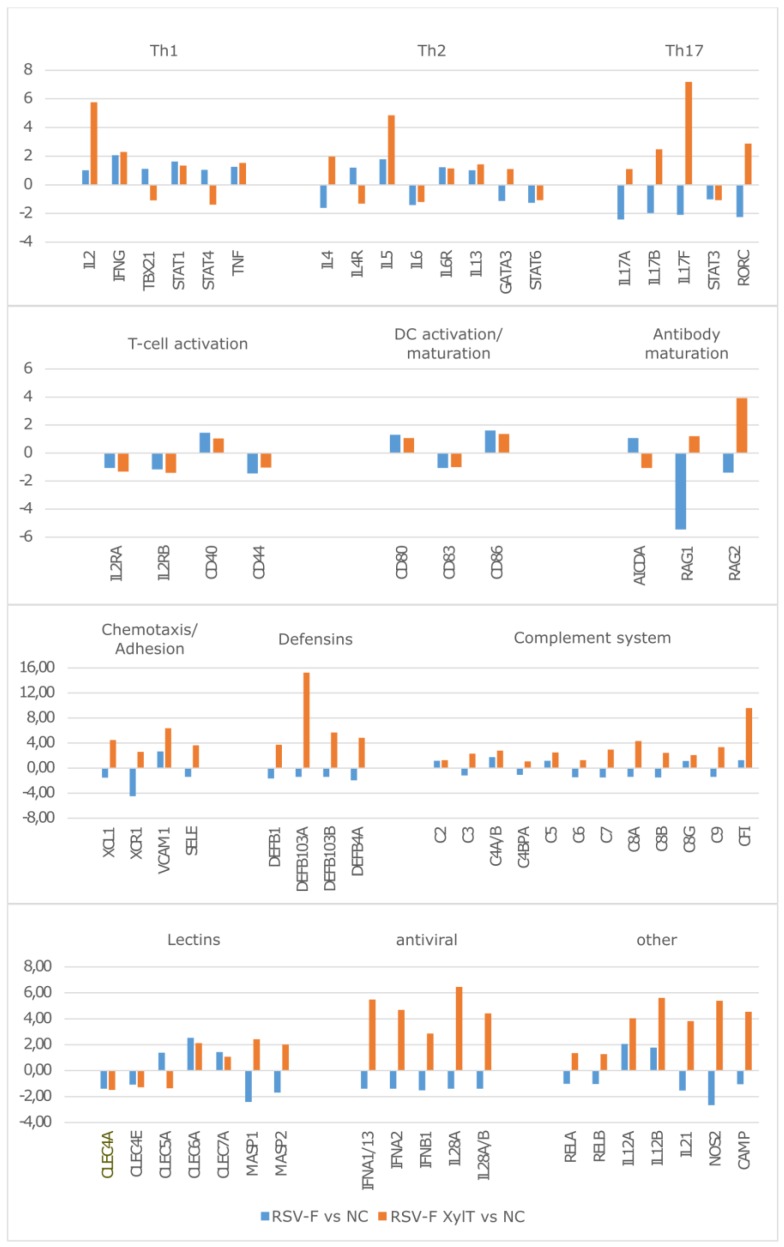
Gene expression ratios of PBMC stimulated for 48 h with RSV-F and RSV-F Xyl+ and maturated DC. Log2-fold changes are shown. NC—negative control without RSV-F treatment.

Gene expression ratios for cells stimulated with RSV-F and RSV-F Xyl+ were based on calculating fold changes compared to negative control. Within the clusters of functionally ordered genes, which are related to the activation of the different T-cell populations, the key cytokines are significantly more activated by RSV-F Xyl+ (Figure 6). More precisely, IL-2 is expressed in much higher amounts within the Th1 subset, IL-4 and IL-5 are strongly upregulated in the Th2 subset and IL-17-related genes are upregulated in the TH17 subset, while the same genes are down-regulated by RSV-F treatment. However, the genes of the related STAT signaling pathways (STAT1, STAT3, STAT4, STAT6) are mostly unaffected at the analyzed time-point, as well as genes of T-cell (CD40, CD44) and DC (CD80, CD83 and CD86) activation. Overall concentrations of Th1, Th2 and Th17 key cytokines were relatively low. Probably, these cytokines are mostly bound to their respective receptors. The expression of those receptors (e.g., IL2RA and B, IL4R) is slightly downregulated. This might indicate a sufficient activation, which is already mitigated (weakened) by decreased receptor expression. Furthermore, gene expression of pro-inflammatory IFNγ and TNF is relatively low, but strong IL17 up-regulation works synergistically to these two cytokines [33].

Genes related to antibody maturation show diverse regulation pattern: while the expression of AICDA—a key player in somatic hypermutation and immune globulin class switch—is not significantly altered, RAG 1 and RAG2 (Recombination Activation Genes 1 and 2) are upregulated by stimulation with RSV-F Xyl+, indicating the onset of antibody maturation, but downregulated by RSV-F treatment. Next to the activation of cellular and humoral components by RSV-F Xyl+, we recognized the activation of entire components of the innate immune systems, like defensins or the complement system. The latter one seems to be activated via the lectin pathway by the mannose binding lectins, MASP1 and MASP2. The role of C-type lectin receptors in activation of murine DC by stimulation with core β1,2-xylosylated glycoprotein was investigated by Brzezicka et al. [34]. They report enhanced uptake, presentation and subsequent T-cell activation by artificially glycosylated ovalbumin; however, xylosylated glycans did not significantly enhance the activation potential in this experimental setup. In our experiments the broad activation of antimicrobial defensins and complement system is complemented by the activation of genes with antiviral functions like IFNα, IFNβ or IL28. Thus, RSV-F Xyl+ seem to initiate a general activation of the immune system while RSV-F does not. Expression patterns of RELA and RELB, which are parts of the NFκB complex, as well as the proinflammatory factors NOS2, CAMP (coding for the multifunctional Cathelicidin Antimicrobial Peptide) or IL21 underline the immune activation by RSV-F Xyl+.

## 4. Conclusions

In this study we successfully glyco-engineered CHO cells for the production of xylosylated *N*-glycans. *Nicotiana tabacum* XylT was functionally expressed in a mammalian cell system, where it was localized in the correct Golgi compartment, had access to endogenous UDP-xylose and could transfer xylose to the *N*-glycan core of a recombinant, co-expressed viral glycoprotein. We were able to show that xylosylated *N*-glycans had strong adjuvant effects in a well-established human organoid immune model, indicating the usefulness of this approach for in vivo models and for vaccination strategies in general. Furthermore, glyco-engineering of CHO cells by the methodology presented here could be extended to other glycan structures with antigenic and therefore adjuvant potency—for example, α1,3-linked core-fucose natively occurring in plant and insect *N*-glycans [5,6,13], or the non-human sialic acid *N*-glycolylneuraminic acid [35].
